# Fast‐food meal reduces peripheral artery endothelial function but not cerebral vascular hypercapnic reactivity in healthy young men

**DOI:** 10.14814/phy2.13867

**Published:** 2018-09-17

**Authors:** Jordan C. Patik, Wesley J. Tucker, Bryon M. Curtis, Michael D. Nelson, Aida Nasirian, Suwon Park, Robert M. Brothers

**Affiliations:** ^1^ Department of Kinesiology The University of Texas at Arlington Arlington Texas; ^2^ College of Nursing and Health Innovation The University of Texas at Arlington Arlington Texas

**Keywords:** Atherosclerosis, endothelial function, human, postprandial, Western diet

## Abstract

Consumption of a representative fast‐food meal (FFMeal) acutely impairs peripheral conduit artery vascular function; however, the effect on cerebral vascular function remains unknown. This study tested the hypothesis that a FFMeal would impair cerebral vascular function as indexed by an attenuated increase in cerebral vascular conductance (CVCI) in the middle cerebral artery (MCA) during a hypercapnic challenge. Ten healthy men (age: 24 ± 3 years, BMI: 24.3 ± 3.8 kg/m^2^) were studied under two conditions; a standardized FFMeal (990 kcals, 50% fat, 36% carbohydrate, 14% protein, and 2120 mg sodium) and a fasting control condition. Basal hemodynamics, cerebral vasomotor reactivity (CVMR), and brachial artery flow‐mediated dilation (BA FMD) were completed after an overnight fast (Pre) and again 2 h and 4 h later both days. To assess CVMR, subjects rebreathed from a 5‐L bag while MCA velocity (MCAV_mean_) was measured using transcranial Doppler (TCD) ultrasound and converted into CVCI (MCAV_mean_/mean arterial pressure). Peripheral artery endothelial function was assessed via BA FMD following a standard 5‐min occlusion protocol. As expected, BA FMD was reduced at 2 h (Pre: 6.6 ± 1.7% vs. 5.2 ± 1.8%, *P* = 0.01). However, despite significant impairment in BA FMD, neither peak CVCI_%baseline_ nor CVMR was affected by the FFMeal (Control–Pre: 1.9 ± 1.1, 2 h: 2.1 ± 1.1, 4 h: 1.7 ± 1.1 ∆CVCI%·∆P_ET_CO_2_^−1^ vs. FFMeal–Pre: 2.1 ± 1.1, 2 h: 2.2 ± 0.7, 4 h: 1.9 ± 0.9 ∆CVCI%·∆P_ET_CO_2_^−1^, time × condition *P* = 0.88). These results suggest that cerebral vascular reactivity to hypercapnia in healthy young men is not altered by an acute FFMeal.

## Introduction

The nutritional profile of a typical fast‐food meal (FFMeal) is conducive to endothelial dysfunction and, as such, consumption of a FFMeal has repeatedly been shown to acutely impair conduit artery vasodilatory function assessed by brachial artery flow‐mediated dilation (BA FMD) (Plotnick et al. 1997; Vogel et al. 1997; Bae et al. 2001; Tsai et al. 2004; Padilla et al. 2006; Tucker et al. 2018). This reduction in endothelial function is attributed to increased production of reactive oxygen species (ROS) due to mitochondrial oxidation of free fatty acids (Wallace et al. 2010) that, in turn, scavenge nitric oxide (NO) and impair endothelium‐mediated dilation (Plotnick et al. 1997; Bae et al. 2001; Tsai et al. 2004). NO, in addition to its profound role in vasodilation, is also antiatherogenic with antithrombotic and anti‐inflammatory effects (Landmesser et al. 2004). Thus, the finding that a single FFMeal impairs NO bioavailability suggests that chronic consumption of similar foods may contribute to the development of atherosclerosis and other cardiovascular disease via decreased NO bioavailability and its aforementioned beneficial properties. Indeed, regular consumption of fast food is associated with an increased risk of developing obesity, type 2 diabetes, cardiovascular disease, and stroke (Pereira et al. 2005; Morgenstern et al. 2009; Odegaard et al. 2012).

Whether a FFMeal has similar deleterious effects on cerebral vascular function is still unclear. The cerebral vasculature is highly sensitive to changes in arterial carbon dioxide concentration (P_a_CO_2_). Under normal conditions, hypercapnia induces vasodilation and thus increases cerebral blood flow (CBF), whereas hypocapnia causes vasoconstriction thereby reducing CBF (Markwalder et al. 1984; Ide et al. 2003). Interestingly, the cerebral vasodilator response to hypercapnia is blunted following inhibition of nitric oxide synthase (NOS) and is restored following supplementation of L‐arginine, a substrate for NOS (Schmetterer et al. 1997). As such, the relationship between changes in CBF and/or cerebral vascular conductance (CVCI) and P_a_CO_2_ is often utilized as an index of cerebral vascular health much the way BA FMD is utilized to assess the peripheral circulation (Claassen et al. 2009; Hurr et al. 2015). In this regard, cerebral vascular responsiveness to changes in P_a_CO_2_, or cerebral vasomotor reactivity (CVMR), is attenuated in populations with known impairments in endothelial function including individuals with carotid artery disease (Gur et al. 1996; Markus and Cullinane 2001), diabetes (Kadoi et al. 2003), sickle cell disease (Nur et al. 2009), and hypertension (Lavi et al. 2006) and is a strong predictor of future cerebrovascular events (Gur et al. 1996; Markus and Cullinane 2001).

To our knowledge, the acute effects of a FFMeal on indices of cerebral vascular function in humans remain largely unknown. It has been recently reported that postprandial hyperlipidemia had no impact on cerebral vascular function in a relatively young population (i.e., ~26 years) (Marley et al. 2017). However, in this study measures were only assessed at 4 h postconsumption and there was also no index of peripheral vascular function provided to verify a vascular effect of the meal (Marley et al. 2017).

While multiple mechanisms mediate hypercapnia‐induced cerebral vasodilation, including a role of prostaglandins and ATP‐sensitive K+ channels (Faraci et al. 1994; Nakahata et al. 2003; Barnes et al. 2012), the partial‐dependence on NO for this response (Iadecola and Xu 1994; Schmetterer et al. 1997; Lavi et al. 2006) makes it susceptible to impairment via NO scavenging by ROS, similar to BA FMD. Accordingly, this study aimed to test the hypothesis that the maximal increase in CVCI and the CVCI/P_a_CO_2_ relationship is blunted following a FFMeal compared to a fasting control condition. To confirm that the FFMeal elicits peripheral vascular dysfunction, we also assessed BA FMD and postocclusion reactive hyperemia, with the hypothesis that acute consumption of the meal would result in attenuated responses. We further hypothesized that all changes in cerebral and peripheral vascular function would be inversely related to augmented serum [TG] following the FFMeal.

## Methods

### Subjects

Ten healthy recreationally active college‐aged men volunteered for participation in the study. Subject characteristics and baseline hemodynamic values are listed in Table 1. All subjects were nonobese according to BMI and waist circumference. All participants were nonsmokers, free of overt cardiovascular, metabolic, and neurological disease, and were not taking any prescription medications or supplements with known antioxidant properties. Participants abstained from caffeine, alcohol, and exercise for a minimum of 24 h prior to each laboratory visit. Furthermore, participants were instructed to consume the same meal for dinner the night prior to each study visit to minimize the impact of a prior meal on basal physiological measures. Women were excluded from the study based upon reports indicating that their vasculature is protected from the insult of an acute FFMeal (Harris et al. 2012). The Institutional Review Board at the University of Texas at Arlington approved all study procedures including the consent process in accordance with the Declaration of Helsinki. All volunteers were given a verbal description of the study procedures and were notified of the potential risks involved prior to providing their informed written consent.

**Table 1:** Subject characteristics

*N*	10
Age (y)	24 ± 3
Height (cm)	176.2 ± 7.6
Weight (kg)	79.1 ± 17.8
BMI (kg·m^−2^)	24.3 ± 3.8
Waist (cm)	82.9 ± 8.5
SBP (mmHg)	116 ± 8
DBP (mmHg)	66 ± 6

All data displayed as Mean ± SD. SBP, Systolic blood pressure; DBP, diastolic blood pressure.

### Protocol

Participants visited the laboratory on two separate occasions separated by at least 3 days. Each visit was randomly assigned to either a FFMeal + water condition or a water‐only fasting condition that served as a time control (control). Studies were conducted at 7:00 am after an overnight fast (>10 h). Upon arrival to the laboratory, participants assumed a supine position on the patient bed for 30 min to allow for equilibration. All data collection was performed in a temperature‐controlled laboratory (~23°C and 40% relative humidity). Baseline (Pre) measurements of basal hemodynamic as well as peripheral and cerebral vascular function were conducted. A blood sample was then collected via venipuncture from an antecubital vein for analysis of serum glucose and lipids. Upon completion of Pre measurements, subjects were given the FFMeal + water or water only. All measurements were repeated at 2 and 4 h following consumption of the FFMeal + water or the water only. The 2‐ and 4‐h data collection time points were each preceded by the same 30 min supine equilibration period. A 4‐h venous blood draw was not repeated to avoid unnecessary subject discomfort. Serum TGs, following a single meal, are known to peak at 2–4 h and stay elevated for up to 6 h (Schneeman et al. 1993).

For each FFMeal trial, the food was purchased from the same fast‐food restaurant and consisted of an Egg McMuffin^®^, a Sausage McMuffin^®^, and two hash browns (McDonald's Corporation, Oak Brook, IL). This meal has been previously reported to elevate serum triglyceride (TG) and blunt brachial artery BA FMD (Plotnick et al. 1997; Vogel et al. 1997; Tsai et al. 2004; Padilla et al. 2006; Johnson et al. 2011). The 990‐kcal meal contained 55 g of total fat (50% of total energy and 19 g of saturated fat), 89 g carbohydrate (36%), 35 g protein (14%), and 2120 mg of sodium. Participants consumed the meal and a 591‐mL bottle of water within 15 min. During the fasting control condition, participants consumed just the bottled water within the same amount of time. The order of the trials was randomized for each participant. Four subjects completed the FFmeal condition first, whereas six subjects completed the control condition first.

### Cerebral vascular function measurement

Cerebral vascular function was determined by transcranial Doppler (TCD) measurement of middle cerebral artery mean velocity (MCAV_mean_) during rebreathing‐induced hypercapnia (Claassen et al. 2007; Hurr et al. 2015). A 2‐MHz TCD probe (Neurovision TOC, Multigon Industries Inc., Yonkers, NY) was placed on the left temple between the eye and ear, superior to the zygomatic arch, and attached via a headband. The TCD signal was optimized by adjusting the probe angle and insonation depth settings. Probe location and settings were noted and used for all subsequent trials. Subjects were then fitted with a tight‐fitting facemask (V2, Hans Rudolph, Shawnee, KS) that was attached to a 5‐L rubber bag for rebreathing. A three‐way stopcock (Hans Rudolph, Shawnee, KS) between the mask and the valve allowed for an instantaneous switch from room air to rebreathing from the bag that had been prefilled with the participant's expired air. End‐tidal CO_2_ tension (P_ET_CO_2_), a proxy for P_a_CO_2_, was measured continuously via a sample line connecting the mouthpiece to a capnograph (Capnocheck Plus, Smiths Medical, Dublin, OH). Arterial oxygen saturation (S_p_O_2_) was monitored throughout the protocol with a digital pulse oximeter (Capnocheck Plus, Smiths Medical, Dublin, OH). Heart rate (HR) and cardiac rhythm were continually assessed via a standard three‐lead ECG (Cardio Card, Nasiff Associates, Central Square, NY). Finger photoplethysmography (Finometer Pro, Finapres Medical Systems, Enschede, NL) was used to assess beat‐to‐beat mean arterial pressure (MAP) after being calibrated to brachial blood pressure measured with an automatic electrosphygnomanometer (Tango M2, Suntech Medical Inc, Morrisville, NC). Stroke volume was derived from the finger photoplethysmograph using the Modelflow method (Wesseling et al. 1993) and used to estimate changes in cardiac output ($\dot{Q}$) and total vascular conductance (TVC = $\dot{Q}$/MAP). Respiratory excursions were monitored with a respiratory belt (Model 1132 Pnuemotrace II, UFI, Morro Bay, CA) placed around the abdomen.

Following instrumentation, participants breathed room air for 6 min of baseline data collection, while MCAV_mean_, MAP, HR, and P_ET_CO_2_ were continuously monitored. After this baseline period the participants then performed the rebreathing protocol as previously described (Hurr et al. 2015). Briefly, the Y‐valve of the three‐way stopcock was switched so they then expired into and inspired from the 5‐l bag. Upon ~2 min of rebreathing, the valve was once again switched allowing the participants to breathe room air for a recovery period. Medical grade oxygen was bled into the bag in order to maintain constant arterial normoxia (S_p_O_2_ = ~97%) during the rebreathing period (Claassen et al. 2007; Brothers 2014; Hurr et al. 2015).

All MAP, HR, MCAV_mean_, and P_ET_CO_2_ data were collected at 400 Hz using a data acquisition system (Powerlab, ADInstruments, Colorado Springs, CO) and stored on a personal computer for offline analysis (Labchart, ADInstruments, Colorado Springs, CO). Average values for P_ET_CO_2_, MCAV_mean_, MAP, HR, and CVCI were determined over the 6 min of baseline and then on a breath‐by‐breath basis during rebreathing. The percent change in CVCI from baseline was determined and the absolute change in P_ET_CO_2_ over the entire rebreathing protocol was assessed. Peak CVCI achieved during rebreathing, CVCI at predetermined stages (i.e., ΔP_ET_CO_2_ of 5, 10, and 15 mmHg), and the linear slope of the increase in CVCI per mmHg ΔP_ET_CO_2_ were analyzed for assessment of CVMR.

### Peripheral vascular function measurement

To verify that the FFMeal impairs endothelial function in our participants, BA FMD was assessed at Pre, 2 h, and 4 h just prior to the cerebral vascular test and following published guidelines (Harris et al. 2010; Thijssen et al. 2011). A Doppler ultrasound (Logiq P5, GE Healthcare, Chicago, IL) with an adjustable frequency (7–12 MHz) linear array transducer was used to image the brachial artery 5–10 cm proximal to the antecubital crease. The probe was held in place via a custom stereotactic clamp and machine settings were adjusted to optimize the B‐mode ultrasound image so that a clear delineation could be made between the lumen and arterial wall. Blood velocity was determined via pulsed wave Doppler set at 5 MHz and duplex video was recorded on a separate computer for later analysis. The angle of insonation was maintained at 60°. Edge detection software (CardioSuite, Quipu, Pisa, IT) was used to continuously measure brachial artery diameter and blood velocity. Diameter measurements were taken across a region of interest with clearly defined upper and lower arterial walls. Second‐by‐second mean blood velocity was calculated as the area of the Doppler spectra and subsequently used for calculation of blood flow (Flow = *π*r^2^*V_mean_*60) and arterial shear rate (sec^−1^ = 4*V_mean_/*D*).

A 10 cm pneumatic cuff (Rapid Cuff Inflation System, D.E. Hokanson Inc, Bellvue, WA) was placed just distal to the medial epicondyle. After 2 min of baseline data collection, the cuff was inflated to 220 mmHg for 5 min. An additional 3 min of video was recorded upon cuff release for analysis of reactive hyperemia and subsequent vasodilation. BA FMD % was defined as the postocclusion maximal change in artery diameter divided by baseline diameter and multiplied by 100. Shear rate area under the curve (AUC) was assessed from cuff release to the point of maximal dilation.

### Blood glucose and lipid assessment

Blood was drawn via venipuncture into serum separator tubes at the Pre and 2‐h time points. After centrifugation, serum was analyzed for glucose and lipids within 24 h at a local laboratory (Laboratory Corporation of America, Burlington, NC).

### Statistical analysis

Based upon previously published findings of impairment in BA FMD following the same FFMeal (Vogel et al. 1997; Padilla et al. 2006; Johnson et al. 2011; Harris et al. 2012), we established that 10 participants would adequately power us to detect differences in BA FMD between conditions. Baseline hemodynamics, blood metabolic parameters, and cerebral vascular reactivity were analyzed using two‐way repeated measures analysis of variance (ANOVA) with the factors condition x time as repeated measures. To assess BA FMD while controlling for the total shear stimulus induced by the reactive hyperemia, analysis of covariance (ANCOVA) was used with shear rate AUC to peak dilation used as a covariate. Where indicated, Tukey's post hoc tests were performed. All statistics were calculated using commercially available software (SPSS Version 22.0, IBM Corp, Armonk, NY; Prism 7, GraphPad Software Inc, La Jolla, CA). Alpha was set at *P* = 0.05.

## Results

### Blood metabolic parameters

As displayed in Table 2, there were no differences between conditions at baseline for blood serum parameters. As expected, serum triglyceride concentration was significantly increased 2 h postprandial. HDL and LDL were each decreased 2 h following the meal while VLDL was increased by the meal. Total cholesterol was unchanged across time or condition. There was not a significant time × condition interaction for serum glucose concentration; however, there was a time‐dependent reduction in glucose.

**Table 2:** Blood serum parameters

	Condition	Time		ANOVA		
		Pre	2 h	Time	Condition	Interaction
Triglycerides (mg·dL^−1^)	Control	112 ± 62	101 ± 54	0.001	0.002	<0.001
	FFMeal	110 ± 62	180 ± 75^a^^,^ b ^,^ c			
HDL (mg·dL^−1^)	Control	47 ± 10	48 ± 11^a^	0.14	0.01	0.02
	FFMeal	44 ± 8	42 ± 9^b^^,^ c			
LDL (mg·dL^−1^)	Control	98 ± 25	102 ± 23	0.12	0.32	0.003
	FFMeal	99 ± 22	90 ± 26^a^^,^ b ^,^ c			
VLDL (mg·dL^−1^)	Control	22 ± 13	20 ± 11	0.001	0.002	<0.001
	FFMeal	22 ± 13	36 ± 15^a^^,^ b ^,^ c			
TC (mg·dL^−1^)	Control	167 ± 28	170 ± 27	0.08	0.78	0.78
	FFMeal	166 ± 26	168 ± 25			
Glucose (mg·dL^−1^)	Control	90 ± 7	87 ± 5	0.003	0.03	0.17
	FFMeal	89 ± 6	80 ± 9			

All data displayed as Mean ± SD. TC, total cholesterol; LDL, low‐density lipoprotein; HDL, high‐density lipoprotein; VLDL, very low‐density lipoprotein. ^a^Significantly different than Pre FFMeal (*P* < 0.05); ^b^Significantly different than Pre control (*P* < 0.05); ^c^Significantly different than same time across condition (*P* < 0.05).

### Basal peripheral hemodynamics

The FFMeal resulted in a significant reduction in diastolic BP and MAP 2 h postprandial that was accompanied by an augmented resting HR (Table 3). Total vascular conductance (TVC) was similarly reduced at 2 h in the FFMeal condition but not during the fasting control. Also presented in Table 3, brachial artery diameter was maintained after the meal while brachial artery blood velocity and thus blood flow were reduced in both conditions after Pre measurements (main effect of time *P* = 0.003 and *P* = 0.01, respectively), with no difference between conditions (time × condition *P* = 0.22 and *P* = 0.30, respectively).

**Table 3:** Hemodynamics

	Condition	Time			ANOVA		
		Pre	2‐h	4‐h	Time	Condition	Interaction
Basal peripheral variables							
SBP (mmHg)	Control	116 ± 5	121 ± 6	121 ± 7	0.01	0.53	0.75
	FFMeal	116 ± 8	118 ± 5	120 ± 7			
DBP (mmHg)	Control	66 ± 7	68 ± 6	70 ± 6	0.01	0.02	0.007
	FFMeal	66 ± 6	59 ± 7^a^^,^ b ^,^ c	67 ± 8			
MAP (mmHg)	Control	83 ± 5	85 ± 5	84 ± 4	0.44	0.05	0.01
	FFMeal	82 ± 5	79 ± 5^c^^,^ e	83 ± 7			
HR (b·min^−1^)	Control	56 ± 6	56 ± 7	56 ± 6	0.07	0.15	0.004
	FFMeal	55 ± 6	61 ± 7^a^^,^ b ^,^ c ^,^ e	58 ± 9			
$\dot{Q}$ (l·min^−1^)	Control	6.1 ± 1	6.0 ± 1	6.2 ± 1	0.74	0.54	0.14
	FFMeal	6.1 ± 1	6.6 ± 1	6.1 ± 1			
TVC (l·min^−1^·mmHg^−1^)	Control	0.075 ± 0.016	0.070 ± 0.015	0.074 ± 0.009	0.31	0.23	0.03
	FFMeal	0.075 ± 0.014	0.084 ± 0.015^c^	0.075 ± 0.014			
BA diameter rest (mm)	Control	4.0 ± 0.5	4.0 ± 0.6	3.9 ± 0.5	0.27	0.90	0.68
	FFMeal	4.0 ± 0.6	3.9 ± 0.6	3.9 ± 0.6			
BA velocity rest (cm·sec^−1^)	Control	25.2 ± 7.9	15.4 ± 6.3	14.6 ± 4.9	0.003	0.91	0.22
	FFMeal	21.8 ± 11.8	15.2 ± 6.2	17.6 ± 5.9			
BA flow rest (mL·min^−1^)	Control	191 ± 92	118 ± 58	110 ± 57	0.007	0.88	0.30
	FFMeal	165 ± 111	111 ± 55	133 ± 80			
Cerebral vascular variables							
MCAV_mean_ rest (cm·sec^−1^)	Control	66.3 ± 8.9	64.7 ± 7.9	63.7 ± 10.3	0.94	0.80	0.34
	FFMeal	62.9 ± 7.9	65.2 ± 12	65.2 ± 10.4			
CVCI rest (cm·sec^−1^·mmHg^−1^)	Control	0.80 ± 0.11	0.76 ± 0.10	0.76 ± 0.16	0.62	0.38	0.05
	FFMeal	0.77 ± 0.12	0.83 ± 0.17	0.79 ± 0.15			
P_ET_CO_2_ rest (mmHg)	Control	41.1 ± 1.1	40.2 ± 2.0	40.0 ± 2.4	0.25	0.08	0.003
	FFMeal	40.4 ± 1.3	42.6 ± 1.6^a^^,^ c ^,^ e	41.6 ± 1.8			
SpO_2_ rest (%)	Control	96.3 ± 2.0	97.2 ± 1.0	97.5 ± 0.3	0.07	0.29	0.86
	FFMeal	96.1 ± 1.3	96.9 ± 1.6	96.8 ± 1.8			
MCAV_mean_ peak (cm·sec^−1^)	Control	96 ± 16.5	93 ± 13.2	91.5 ± 14	0.29	0.87	0.91
	FFMeal	95.2 ± 13.9	94 ± 19.5	92.2 ± 18.7			
CVCI peak (%Baseline)	Control	136 ± 14	136 ± 14	134 ± 15	0.41	0.24	0.33
	Peak	137 ± 15	130 ± 10	129 ± 6			
P_ET_CO_2_ peak (mmHg)	Control	58.5 ± 1.7	58.3 ± 1.9	58.3 ± 1.6	0.08	0.07	0.02
	FFMeal	59 ± 1.6	60.4 ± 1.4^a^^,^ b ^,^ c ^,^ d ^,^ e	59 ± 2.1			
SpO_2_ peak (%)	Control	95.9 ± 2.8	97.6 ± 0.8	97.7 ± 1.2	0.01	0.28	0.34
	FFMeal	96.2 ± 2.0	96.9 ± 1.4	97.1 ± 0.9			

All data displayed as Mean ± SD. SBP: Systolic Blood Pressure, DBP: Diastolic Blood Pressure, MAP: Mean Arterial Pressure, HR: Heart Rate, $\dot{Q}$: Cardiac Output, TVC: Total Vascular Conductance, MCAV_mean_; Mean Middle Cerebral Artery Velocity, CVCI; Cerebral Vascular Conductance Index, P_ET_CO_2_: End‐Tidal Carbon Dioxide Pressure, S_P_O2: Peripheral Oxygen Saturation, BA; Brachial Artery. ^a^Significantly different than Pre FFMeal (*P *<* *0.05); ^b^Significantly different than Pre control (*P *<* *0.05); ^c^Significantly different than same time across condition (*P *<* *0.05); ^d^Significantly different than 4 h within condition (*P *<* *0.05); ^e^Significantly different than control 4 h (*P *<* *0.05).

### Basal cerebral hemodynamics

The FFMeal resulted in an increase in basal P_ET_CO_2_ at 2 h (vs. Pre: *P* = 0.01, vs. control 2 h, *P* = 0.01, Table 3). Resting MCAV_mean_ was not affected by the meal (time × condition *P* = 0.34, Table 3). Basal CVCI was not different across time or condition (Table 3). Taken together, these data suggest that cerebral perfusion is maintained after a FFMeal despite an attenuated MAP.

### Cerebral vascular function

**Figure 1:**
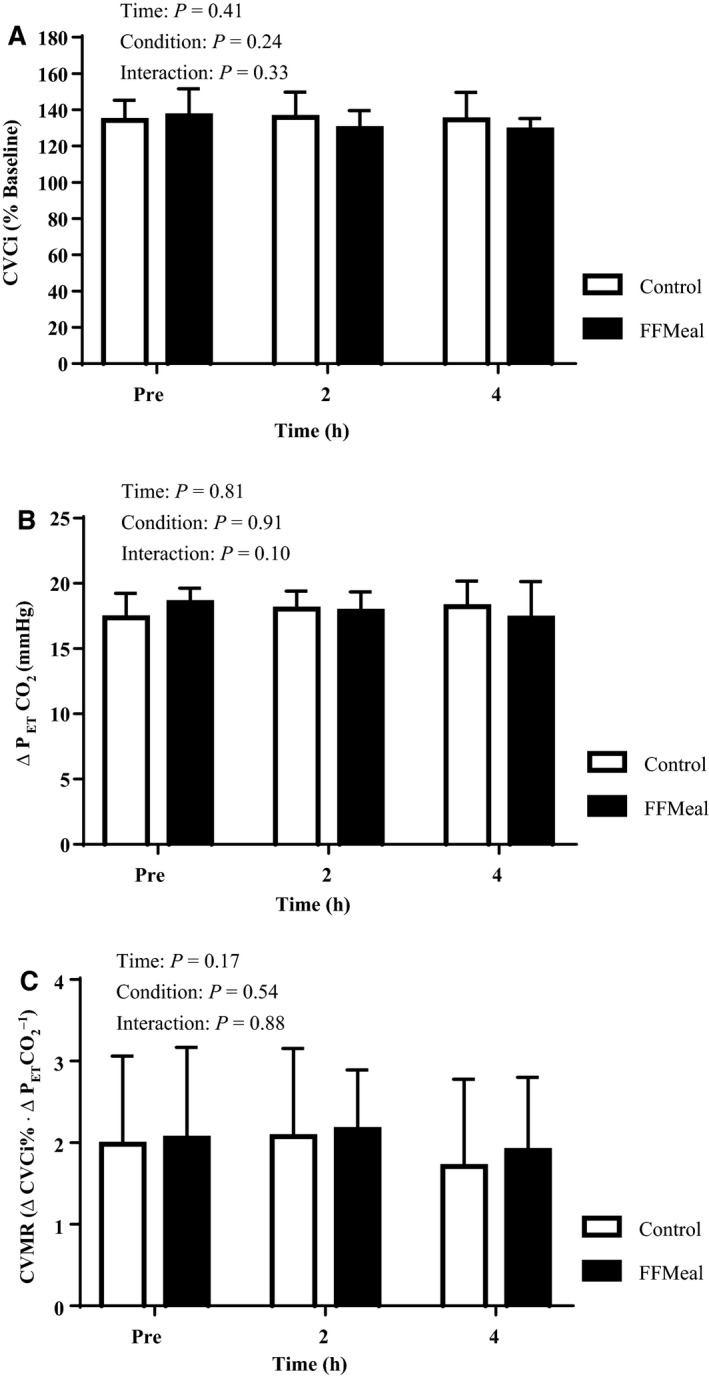
(A) Peak CVCI (% Baseline) at Pre, 2 h, and 4 h in control (open bars) and FFMeal (solid bars) conditions. No significant differences were detected across time or condition (interaction: *P* = 0.33). (B) ∆P_ET_CO_2_ from resting levels at peak CVCI indicating that peak CVCI occurs at a consistent ∆P_ET_CO_2_ (time x condition: *P* = 0.10). (C) There was no difference in reactivity (∆CVCI·∆P_ET_CO_2_^−1^) across time or condition (*P* = 0.88). All data are expressed as Mean ± SD.

Peak MCAV was not different across time or condition (interaction: *P* = 0.91, Table 3). Likewise, there was no effect of FFMeal on peak cerebral conductance (time × condition *P* = 0.33, Table 3 and Fig. 1A). As shown in Figure 1B, peak CVCI_%baseline_ occurred at the same ΔP_ET_CO_2_ (*P* = 0.10), however, this P_ET_CO_2_ was achieved at a higher absolute level (Table 3, FFMeal 2 h versus FFMeal Pre: *P* = 0.02, FFMeal 2 h vs. Control 2 h: *P* < 0.001) at 2 h due to the elevated postprandial baseline P_ET_CO_2_. There was no significant effect of the meal across time when data were analyzed at ΔP_ET_CO_2_ of 5, 10, and 15 mmHg (not shown, time × condition *P* = 0.37, *P* = 0.91, and 0.51, respectively). The slope of the increase in CVCI_%baseline_ for a given change in P_ET_CO_2_ was not different across time or condition (Fig. 1C, FFMeal: Baseline – 2.1 ± 1.1, 2 h – 2.2 ± 0.7, 4 h – 1.9 ± 0.9 ΔCVCI_%baseline_·Δ P_ET_CO_2_^−1^; Control: Baseline – 2.0 ± 1.1, 2 h – 2.1 ± 1.1, 4 h – 1.7 ± 1.1 ΔCVCI_%baseline_·Δ P_ET_CO_2_^−1^, time × condition *P* = 0.88). Furthermore, there was no significant relationship with the change in serum [TG] and peak CVCI_%baseline_ or slope of CVCI relative to ΔP_ET_CO_2_ at 2 h or 4 h (not shown, all *P* > 0.47).

### Peripheral vascular function

**Figure 2:**
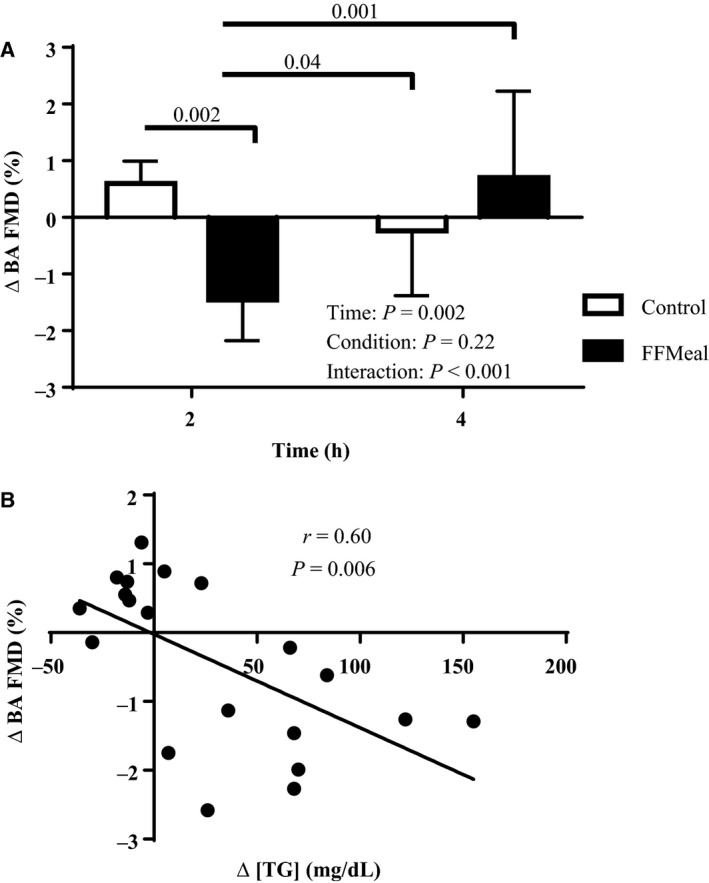
(A) The change in BA FMD from Pre at 2 h and 4 h in control (open bars) and FFMeal (solid bars) conditions. Following FFMeal, BA FMD was reduced at 2 h compared to control. All data are expressed as Mean ± SD. (B) The relationship between the change in serum [TG] and BA FMD from Pre to 2 h indicating a reduction in BA FMD with an increase in serum [TG].

**Figure 3:**
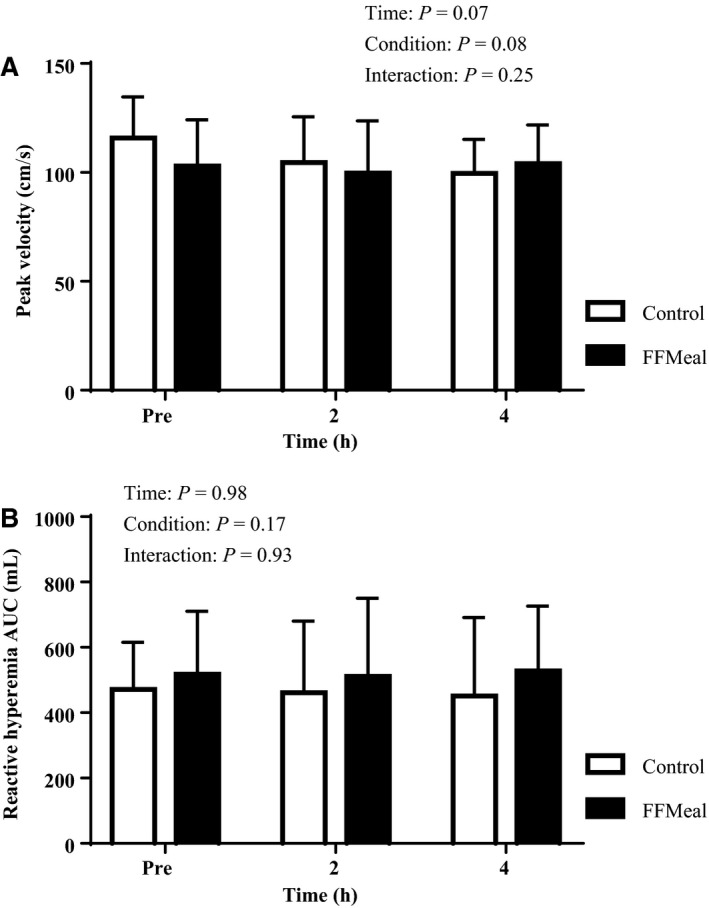
(A) Peak reactive hyperemia blood velocity in the brachial artery following 5 min of occlusion in control (open bars) and FFmeal (solid bars). (B) Reactive hyperemia AUC (i.e., total blood flow) during the first 120 sec post cuff release. All data are expressed as Mean ± SD. Neither time nor condition significantly affected either index of peripheral microvascular function.

BA FMD responses were analyzed with Shear AUC as a covariate to account for the shear stimulus to the point of peak dilation. Intra‐ and interday reliability coefficients of variance were 11.8% and 12.7%, respectively. Figure 2A illustrates the change in BA FMD %, from Pre, at 2‐h and 4‐h time points for both conditions. ∆ BA FMD % was reduced at 2 h following FFMeal compared to both 2 h and 4 h in control condition and 4 h following FFMeal. Figure 2B illustrates that the change in BA FMD at 2 h was inversely correlated with the change in [TG] at 2 h (*r* = 0.60, *P* = 0.01). The effect of the FFMeal on peripheral microvascular function was assessed by the hyperemic responses following cuff release. There was no effect of the FFMeal on peak blood velocity (Fig. 3A), hyperemic blood flow AUC in excess of baseline for 120 sec following cuff release (Fig. 3B), or peak blood flow (time × condition *P* = 0.75, not shown).

## Discussion

The primary finding of this study is that, contrary to our hypothesis, a FFMeal did not acutely reduce basal CVCI or impair cerebral vascular function during a hypercapnic rebreathing challenge. In agreement with previous literature (Plotnick et al. 1997; Vogel et al. 1997), we observed a transient reduction in brachial artery BA FMD that was related to an increase in serum TG. Therefore, this study indicates that a single FFMeal results in a transient impairment in peripheral conduit artery endothelial function; however, it appears that CVMR to hypercapnia in the cerebral circulation of healthy young men is not acutely impaired by a FFMeal.

### Postprandial cerebral vascular reactivity

Marley and colleagues recently reported that postprandial hyperlipidemia results in blunted cerebral vascular reactivity to hypercapnia in older men (>60 year) while it has no deleterious effects in young men (<35 year) (Marley et al. 2017). A time control experiment and confirmation that their meal condition did, in fact, impair peripheral vascular function was not provided in that study (Marley et al. 2017). This study addressed these issues via our crossover design and use of BA FMD to assess peripheral conduit artery vasodilation. While the meal used in this study had a slightly different nutrient profile, we similarly observed maintained cerebral vascular reactivity to hypercapnia in young men despite clear evidence of attenuated BA FMD consistent with previous literature (Plotnick et al. 1997; Vogel et al. 1997; Tsai et al. 2004; Padilla et al. 2006).

The reduction in BA FMD observed previously is attributed to a TG‐induced increase in systemic ROS, which then scavenges NO (Plotnick et al. 1997; Bae et al. 2001; Tsai et al. 2004). Although our meal elicited attenuations in NO‐mediated peripheral artery vasodilation at 2 h, we did not observe a reduction in CVMR at 2 or 4 h postprandial in this study. While this observation opposed our hypothesis, a further examination of the role of NO in CVMR may provide insight to this finding. Rodent studies have established that cerebral vascular CO_2_ reactivity is highly NO dependent. For instance, Iadecola demonstrated that cerebral blood flow responses to hypercapnia abolished after NOS inhibition and restored when L‐Arginine was infused concurrently (Iadecola 1992). In primates, however, NOS inhibition blunts global cerebral blood flow at rest, but only attenuated hypercapnic dilation by ~30% in the cortex, with no reductions in blood flow to other regions (McPherson et al. 1995). Data in humans on the role of NO in cerebral vascular CO_2_ reactivity are mixed. Schmetterer and colleagues demonstrated that intravenous N^G^‐monmethyle‐l‐arginine (L‐NMMA) blunted basal cerebral blood flow and CO_2_ reactivity in the MCA by greater than 40% (Schmetterer et al. 1997). Joshi et al. (2000) confirmed the reductions in basal flow angiographically with intracarotid infusions of the same NOS inhibitor, however, they did not test CO_2_ reactivity. Exogenous NO, in the form of sodium nitroprusside, normalizes reduced CVMR in hypertensives, suggesting that this group with known endothelial dysfunction has impaired cerebral vascular production and/or release of NO during hypercapnia (Lavi et al. 2006). However, another study showed no effect of increasing doses of L‐NMMA on hypercapnic hyperemia when assessed by carotid artery ultrasound flow and TCD‐derived blood velocity (White et al. 1998).

Other than NO, prostaglandins (PG) and endothelium‐derived hyperpolarizing factors (EDHF) may be responsible for cerebral vasodilation during hypercapnia. K_ATP_ channel opening contributes to hypercapnic cerebral arteriolar dilation in rabbits (Faraci et al. 1994); however, when tested in humans, K_ATP_ channel inhibitor glibenclamide did not attenuate CVMR (Bayerle‐Eder et al. 2000). The modest CO_2_ stimulus and concurrent insulin infusion used in the study by Bayerle‐Eder et al. (2000) may explain the apparent species differences (Bayerle‐Eder et al. 2000). CVMR in young adults is blunted by 42% after treatment with indomethacin indicating a substantial role for PG in hypercapnia‐induced cerebral vasodilation (Barnes et al. 2012). We speculate that in healthy individuals, other vasodilatory mechanisms can compensate for any reduction in NO bioavailability induced by postprandial hypertriglyceridemia. Consistent with this hypothesis, Marley et al. (2017) observed no reduction in CVMR in young men despite a clear increase in ROS and reduction in a marker of NO bioavailability. In contrast, older men presented with increased oxidative stress at baseline that was unaffected by postprandial hyperlipidemia, yet NO bioavailability and CVMR were clearly reduced following the meal (Marley et al. 2017). This finding may indicate an increased reliance on NO for hypercapnic cerebral vasodilation in aging, thus resulting in an impaired ability of the cerebral vasculature to compensate during a dietary insult via non‐NO pathways. However, more mechanistic studies are needed to better understand these observations.

An alternative interpretation of our findings is that FFMeal did, in fact, attenuate NO bioavailability and our results are due to impaired dilation of the MCA. If dilation of the MCA to hypercapnia (Coverdale et al. 2014; Verbree et al. 2014) was blunted in the meal condition, a reduction in volumetric flow during hypercapnia could be masked by similar changes in MCAV_mean_. To this point, NOS inhibition during normocapnia attenuates ICA volumetric flow without a change in MCAV_mean_, indicating constriction of the MCA (White et al. 1998). As TCD only allows for the measurement of velocity and not volumetric flow, this possibility cannot be ruled out without angiographic or magnetic resonance measurements of MCA diameter.

### Peripheral vascular function

In this study, BA FMD was solely tested to confirm that the FFMeal acutely impaired peripheral endothelial function. The effects of a FFMeal on brachial artery endothelial function have been well described with the majority of studies observing acute reductions in postprandial BA FMD (Plotnick et al. 1997; Vogel et al. 1997; Bae et al. 2001; Padilla et al. 2006; Johnson et al. 2011; Tucker et al. 2018). In this study, BAFMD was significantly decreased 2 h following consumption of the FFMeal, but was restored by 4 h postmeal consistent with the recent study by Tucker and colleagues (2018). These findings confirm that the FFmeal utilized in this study was sufficient to replicate the previous findings of impaired peripheral vascular function, assessed as BA FMD (Plotnick et al. 1997; Vogel et al. 1997; Bae et al. 2001; Padilla et al. 2006; Johnson et al. 2011; Tucker et al. 2018).

The peripheral microvasculature, as assessed by the postocclusion reactive hyperemia was unaffected across time and condition. As reactive hyperemia is mediated by EDHF and largely independent of NO (Crecelius et al. 2013), it is likely less susceptible to ROS relative to conduit artery dilation. In terms of basal blood flow, we observed a main effect of time that indicated attenuations in basal brachial artery blood velocity and flow independent of condition. The reasons for this disparity remain unclear.

### General hemodynamic effects of a fast‐food meal

In this study, we observed a marked decrease in diastolic BP and MAP at 2 h in the FFMeal condition. We speculate that this observation is a product of the significant postprandial attenuation in TPR and the lack of a significant concurrent augmentation in cardiac output (Table 3). The reduced TPR is likely due to splanchnic dilation during digestion (Matheson et al. 2000). While consumption of a mixed meal can induce insulin‐ and/or TG‐mediated peripheral vasodilation (Gokce et al. 2001; Fugmann et al. 2003), the lack of an increase in BA blood flow (Table 3) can be taken as evidence that this was not occurring. Blood flow was not measured in the lower extremities, and thus meal‐related dilation of the leg vasculature cannot be ruled out.

### Limitations

The experimental design of this study has several limitations. This study compared the effects of a FFMeal with a fasting condition rather than an isocaloric control meal. Admittedly, this limits our ability to ascribe the vascular effects of the meal to a specific macro‐ or micronutrient component within the meal. Previous studies have focused on fat content of this meal (Plotnick et al. 1997; Vogel et al. 1997; Tsai et al. 2004; Padilla et al. 2006), however, there is substantial evidence that acute sodium intake (Dickinson et al. 2011) and postprandial hyperglycemia (Akbari et al. 1998) each independently result in blunted endothelium‐dependent vasodilation. Therefore, it is logical to expect greater differences between FFMeal and fasting control compared to a FFMeal and an isocaloric control condition. Likewise, measurements of ROS were not made in this study. However, others have shown that the current test meal (Tsai et al. 2004) and similar meals (Bae et al. 2001; Marley et al. 2017) result in acute increases in systemic ROS, whereas antioxidant supplementation abolishes the meal‐related reductions in FMD (Plotnick et al. 1997).

This study may also be limited by several technical considerations. First, as mentioned previously, TCD only measures blood velocity. It has recently been shown that the MCA dilates during hypercapnia (Coverdale et al. 2014, 2015; Verbree et al. 2014) and therefore we are unable to be certain that MCA diameter and cerebral blood flow responses were unaffected by the high‐fat meal. The use of cerebral MRI or ultrasonic measurement of blood flow in the internal carotid artery could help to solidify these findings. Additionally, we did not induce hypocapnia via hyperventilation, therefore we were only able to assess the vasodilatory range and were unable to model the CVMR response using a four‐parameter logistic equation (Claassen et al. 2007).

## Conclusions

In conclusion, this study observed that basal cerebral vascular reactivity to hypercapnia was not affected by a single fast‐food meal despite an acute attenuation of brachial artery endothelial function. In healthy young men, cerebral vascular responses to hypercapnia likely occur due to a variety of mechanisms in addition to NO and, as such, are protected from the TG‐induced ROS that impair endothelial function in peripheral arteries. Future research is needed to confirm these findings in other populations that have impaired cerebral vascular function.

## Conflict of Interest

The authors have no conflict(s)‐of Interest/Disclosures to report.

## References

[phy213867-bib-0001] Akbari, C. M. , R. Saouaf , D. F. Barnhill , P. A. Newman , F. W. LoGerfo , and A. Veves . 1998 Endothelium‐dependent vasodilatation is impaired in both microcirculation and macrocirculation during acute hyperglycemia. J. Vasc. Surg. 28:687–694. 9786265 10.1016/s0741-5214(98)70095-3

[phy213867-bib-0002] Bae, J.‐H. , E. Bassenge , K.‐B. Kim , Y.‐N. Kim , K.‐S. Kim , H.‐J. Lee , et al. 2001 Postprandial hypertriglyceridemia impairs endothelial function by enhanced oxidant stress. Atherosclerosis 155:517–523. 11254924 10.1016/s0021-9150(00)00601-8

[phy213867-bib-0003] Barnes, J. N. , J. E. Schmidt , W. T. Nicholson , and M. J. Joyner . 2012 Cyclooxygenase inhibition abolishes age‐related differences in cerebral vasodilator responses to hypercapnia. J. Appl. Physiol. 112:1884–1890. 22442028 10.1152/japplphysiol.01270.2011 PMC3379157

[phy213867-bib-0004] Bayerle‐Eder, M. , M. Wolzt , E. Polska , H. Langenberger , J. Pleiner , D. Teherani , et al. 2000 Hypercapnia‐induced cerebral and ocular vasodilation is not altered by glibenclamide in humans. Am. J. Physiol. Regul. Integr. Comp. Physiol. 278:R1667–R1673. 10848537 10.1152/ajpregu.2000.278.6.R1667

[phy213867-bib-0005] Brothers, R. M. 2014 Cerebral vasomotor reactivity: steady‐state versus transient changes in carbon dioxide tension. Exp. Physiol. 99:1499–1510. 25172891 10.1113/expphysiol.2014.081190 PMC4218865

[phy213867-bib-0006] Claassen, J. A. , R. Zhang , Q. Fu , S. Witkowski , and B. D. Levine . 2007 Transcranial Doppler estimation of cerebral blood flow and cerebrovascular conductance during modified rebreathing. J. Appl. Physiol. 102:870–877. 17110510 10.1152/japplphysiol.00906.2006

[phy213867-bib-0007] Claassen, J. A. H. R. , B. D. Levine , and R. Zhang . 2009 Cerebral vasomotor reactivity before and after blood pressure reduction in hypertensive patients. Am. J. Hypertens. 22:384–391. 19229191 10.1038/ajh.2009.2

[phy213867-bib-0008] Coverdale, N. S. , J. S. Gati , O. Opalevych , A. Perrotta , and J. K. Shoemaker . 2014 Cerebral blood flow velocity underestimates cerebral blood flow during modest hypercapnia and hypocapnia. J. Appl. Physiol. 117:1090–1096. 25012027 10.1152/japplphysiol.00285.2014

[phy213867-bib-0009] Coverdale, N. S. , S. Lalande , A. Perrotta , and J. K. Shoemaker . 2015 Heterogeneous patterns of vasoreactivity in the middle cerebral and internal carotid arteries. Am. J. Physiol. Heart Circ. Physiol. 308:H1030–H1038. 25724496 10.1152/ajpheart.00761.2014

[phy213867-bib-0010] Crecelius, A. R. , J. C. Richards , G. J. Luckasen , D. G. Larson , and F. A. Dinenno . 2013 Reactive hyperemia occurs via activation of inwardly rectifying potassium channels and Na/K‐ATPase in humans. Circ. Res. 113:1023–1032. 23940309 10.1161/CIRCRESAHA.113.301675 PMC3871189

[phy213867-bib-0011] Dickinson, K. M. , P. M. Clifton , and J. B. Keogh . 2011 Endothelial function is impaired after a high‐salt meal in healthy subjects. Am. J. Clin. Nutr. 93:500–505. 21228265 10.3945/ajcn.110.006155

[phy213867-bib-0012] Faraci, F. M. , K. R. Breese , and D. D. Heistad . 1994 Cerebral vasodilation during hypercapnia. Role of glibenclamide‐sensitive potassium channels and nitric oxide. Stroke 25:1679–1683. 8042220 10.1161/01.str.25.8.1679

[phy213867-bib-0013] Fugmann, A. , J. Millgard , M. Sarabi , C. Berne , and L. Lind . 2003 Central and peripheral haemodynamic effects of hyperglycaemia, hyperinsulinaemia, hyperlipidaemia or a mixed meal. Clin. Sci. 105:715–721. 12882644 10.1042/CS20030036

[phy213867-bib-0014] Gokce, N. , S. J. Duffy , L. M. Hunter , J. F. Keaney , and J. A. Vita . 2001 Acute hypertriglyceridemia is associated with peripheral vasodilation and increased basal flow in healthy young adults. Am. J. Cardiol. 88:153–159. 11448412 10.1016/s0002-9149(01)01610-1

[phy213867-bib-0015] Gur, A. Y. , I. Bova , and N. M. Bornstein . 1996 Is impaired cerebral vasomotor reactivity a predictive factor of stroke in asymptomatic patients? Stroke 27:2188–2190. 8969778 10.1161/01.str.27.12.2188

[phy213867-bib-0016] Harris, R. A. , S. K. Nishiyama , D. W. Wray , and R. S. Richardson . 2010 Ultrasound assessment of flow‐mediated dilation. Hypertension 55:1075–1085. 20351340 10.1161/HYPERTENSIONAHA.110.150821 PMC2878744

[phy213867-bib-0017] Harris, R. A. , V. Tedjasaputra , J. Zhao , and R. S. Richardson . 2012 Premenopausal women exhibit an inherent protection of endothelial function following a high‐fat meal. Reprod. Sci. 19:221–228. 22383760 10.1177/1933719111418125 PMC3343134

[phy213867-bib-0018] Hurr, C. , K. Kim , M. L. Harrison , and R. M. Brothers . 2015 Attenuated cerebral vasodilatory capacity in response to hypercapnia in college‐aged African Americans. Exp. Physiol. 100:35–43. 25557729 10.1113/expphysiol.2014.082362 PMC4489322

[phy213867-bib-0019] Iadecola, C. 1992 Does nitric oxide mediate the increases in cerebral blood flow elicited by hypercapnia? Proc. Natl Acad. Sci. USA 89:3913–3916. 1570313 10.1073/pnas.89.9.3913 PMC525601

[phy213867-bib-0020] Iadecola, C. , and X. Xu . 1994 Nitro‐L‐arginine attenuates hypercapnic cerebrovasodilation without affecting cerebral metabolism. Am. J. Physiol. 266:R518–R525. 8141411 10.1152/ajpregu.1994.266.2.R518

[phy213867-bib-0021] Ide, K. , M. Eliasziw , and M. J. Poulin . 2003 Relationship between middle cerebral artery blood velocity and end‐tidal PCO2 in the hypocapnic‐hypercapnic range in humans. J. Appl. Physiol. 95:129–137. 19278048 10.1152/japplphysiol.01186.2002

[phy213867-bib-0022] Johnson, B. D. , J. Padilla , R. A. Harris , and J. P. Wallace . 2011 Vascular consequences of a high‐fat meal in physically active and inactive adults. Appl. Physiol. Nutr. Metab. 36:368–375. 21574775 10.1139/H11-028

[phy213867-bib-0023] Joshi, S. , W. L. Young , D. H. Duong , N. D. Ostapkovich , B. D. Aagaard , T. Hashimoto , et al. 2000 Intracarotid infusion of the nitric oxide synthase inhibitor, L‐NMMA, modestly decreases cerebral blood flow in human subjects. Anesthesiology 93:699–707. 10969303 10.1097/00000542-200009000-00019

[phy213867-bib-0024] Kadoi, Y. , H. Hinohara , F. Kunimoto , S. Saito , M. Ide , H. Hiraoka , et al. 2003 Diabetic patients have an impaired cerebral vasodilatory response to hypercapnia under propofol anesthesia. Stroke 34:2399–2403. 12958324 10.1161/01.STR.0000090471.28672.65

[phy213867-bib-0025] Landmesser, U. , B. Hornig , and H. Drexler . 2004 Endothelial function: a critical determinant in atherosclerosis? Circulation 109(21 suppl 1):II–27. 10.1161/01.CIR.0000129501.88485.1f 15173060

[phy213867-bib-0026] Lavi, S. , D. Gaitini , V. Milloul , and G. Jacob . 2006 Impaired cerebral CO2 vasoreactivity: association with endothelial dysfunction. Am. J. Physiol. Heart Circ. Physiol. 291:H1856–H1861. 16766649 10.1152/ajpheart.00014.2006

[phy213867-bib-0027] Markus, H. , and M. Cullinane . 2001 Severely impaired cerebrovascular reactivity predicts stroke and TIA risk in patients with carotid artery stenosis and occlusion. Brain 124:457–467. 11222446 10.1093/brain/124.3.457

[phy213867-bib-0028] Markwalder, T.‐M. , P. Grolimund , R. W. Seiler , F. Roth , and R. Aaslid . 1984 Dependency of blood flow velocity in the middle cerebral artery on end‐tidal carbon dioxide partial pressure—a transcranial ultrasound Doppler Study. J. Cereb. Blood Flow Metab. 4:368–372. 6432808 10.1038/jcbfm.1984.54

[phy213867-bib-0029] Marley, C. J. , D. Hodson , J. V. Brugniaux , L. Fall , and D. M. Bailey . 2017 Post‐prandial hyperlipidaemia results in systemic nitrosative stress and impaired cerebrovascular function in the aged. Clin. Sci. 131:2807–2812. 29054860 10.1042/CS20171406

[phy213867-bib-0030] Matheson, P. J. , M. A. Wilson , and R. N. Garrison . 2000 Regulation of intestinal blood flow. J. Surg. Res. 93:182–196. 10945962 10.1006/jsre.2000.5862

[phy213867-bib-0031] McPherson, R. W. , J. R. Kirsch , R. F. Ghaly , and R. J. Traystman . 1995 Effect of nitric oxide synthase inhibition on the cerebral vascular response to hypercapnia in primates. Stroke 26:682–687. 7535954 10.1161/01.str.26.4.682

[phy213867-bib-0032] Morgenstern, L. B. , J. D. Escobar , B. N. Sánchez , R. Hughes , B. G. Zuniga , N. Garcia , et al. 2009 Fast food and neighborhood stroke risk. Ann. Neurol. 66:165–170. 19743456 10.1002/ana.21726 PMC2745509

[phy213867-bib-0033] Nakahata, K. , H. Kinoshita , Y. Hirano , Y. Kimoto , H. Iranami , and Y. Hatano . 2003 Mild hypercapnia induces vasodilation via adenosine triphosphate‐sensitive K+ channels in parenchymal microvessels of the rat cerebral cortex. Anesthesiology 99:1333–1339. 14639145 10.1097/00000542-200312000-00014

[phy213867-bib-0034] Nur, E. , Y. S. Kim , J. Truijen , E. J. van Beers , S. C. Davis , D. P. Brandjes , et al. 2009 Cerebrovascular reserve capacity is impaired in patients with sickle cell disease. Blood 114:3473–3478. 19700663 10.1182/blood-2009-05-223859

[phy213867-bib-0035] Odegaard, A. O. , W. P. Koh , J.‐M. Yuan , M. D. Gross , and M. A. Pereira . 2012 Western‐style fast food intake and cardiometabolic risk in an Eastern Country. Circulation 126:182–188. 22753304 10.1161/CIRCULATIONAHA.111.084004 PMC4059207

[phy213867-bib-0036] Padilla, J. , R. A. Harris , A. D. Fly , L. D. Rink , and J. P. Wallace . 2006 The effect of acute exercise on endothelial function following a high‐fat meal. Eur. J. Appl. Physiol. 98:256–262. 16896723 10.1007/s00421-006-0272-z

[phy213867-bib-0037] Pereira, M. A. , A. I. Kartashov , C. B. Ebbeling , L. Van Horn , M. L. Slattery , D. R. Jacobs , et al. 2005 Fast‐food habits, weight gain, and insulin resistance (the CARDIA study): 15‐year prospective analysis. Lancet 365:36–42. 15639678 10.1016/S0140-6736(04)17663-0

[phy213867-bib-0038] Plotnick, G. D. , M. C. Corretti , and R. A. Vogel . 1997 Effect of antioxidant vitamins on the transient impairment of endothelium—dependent brachial artery vasoactivity following a single high‐fat meal. JAMA 278:1682–1686. 9388088

[phy213867-bib-0039] Schmetterer, L. , O. Findl , K. Strenn , U. Graselli , J. Kastner , H.‐G. Eichler , et al. 1997 Role of NO in the O2 and CO2 responsiveness of cerebral and ocular circulation in humans. Am. J. Physiol. Regul. Integr. Comp. Physiol. 273:R2005–R2012. 10.1152/ajpregu.1997.273.6.R2005 9435655

[phy213867-bib-0040] Schneeman, B. O. , L. Kotite , K. M. Todd , and R. J. Havel . 1993 Relationships between the responses of triglyceride‐rich lipoproteins in blood plasma containing apolipoproteins B‐48 and B‐100 to a fat‐containing meal in normolipidemic humans. Proc. Natl Acad. Sci. USA 90:2069–2073. 8446630 10.1073/pnas.90.5.2069 PMC46022

[phy213867-bib-0041] Thijssen, D. H. J. , M. A. Black , K. E. Pyke , J. Padilla , G. Atkinson , R. A. Harris , et al. 2011 Assessment of flow‐mediated dilation in humans: a methodological and physiological guideline. Am. J. Physiol. Heart Circ. Physiol. 300:H2–H12. 20952670 10.1152/ajpheart.00471.2010 PMC3023245

[phy213867-bib-0042] Tsai, W.‐C. , Y.‐H. Li , C.‐C. Lin , T.‐H. Chao , and J.‐H. Chen . 2004 Effects of oxidative stress on endothelial function after a high‐fat meal. Clin. Sci. 106:315–319. 14561213 10.1042/CS20030227

[phy213867-bib-0043] Tucker, W. J. , B. J. Sawyer , C. L. Jarrett , D. M. Bhammar , J. R. Ryder , S. S. Angadi , et al. 2018 High‐intensity interval exercise attenuates, but does not eliminate, endothelial dysfunction after a fast‐food meal. Am. J. Physiol. Heart Circ. Physiol. 314:H188–H194. 29101171 10.1152/ajpheart.00384.2017

[phy213867-bib-0044] Verbree, J. , A. S. Bronzwaer , E. Ghariq , M. J. Versluis , M. J. Daemen , M. A. van Buchem , et al. 2014 Assessment of middle cerebral artery diameter during hypocapnia and hypercapnia in humans using ultra‐high‐field MRI. J. Appl. Physiol. 117:1084–1089. 25190741 10.1152/japplphysiol.00651.2014

[phy213867-bib-0045] Vogel, R. A. , M. C. Corretti , and G. D. Plotnick . 1997 Effect of a single high‐fat meal on endothelial function in healthy subjects. Am. J. Cardiol. 79:350–354. 9036757 10.1016/s0002-9149(96)00760-6

[phy213867-bib-0046] Wallace, J. P. , B. Johnson , J. Padilla , and K. Mather . 2010 Postprandial lipaemia, oxidative stress and endothelial function: a review. Int. J. Clin. Pract. 64:389–403. 20456177 10.1111/j.1742-1241.2009.02146.x

[phy213867-bib-0047] Wesseling, K. H. , J. R. Jansen , J. J. Settels , and J. J. Schreuder . 1993 Computation of aortic flow from pressure in humans using a nonlinear, three‐element model. J. Appl. Physiol. 74:2566–2573. 8335593 10.1152/jappl.1993.74.5.2566

[phy213867-bib-0048] White, R. P. , C. Deane , P. Vallance , and H. S. Markus . 1998 Nitric oxide synthase inhibition in humans reduces cerebral blood flow but not the hyperemic response to hypercapnia. Stroke 29:467–472. 9472891 10.1161/01.str.29.2.467

